# The Current Understanding of the Molecular Pathogenesis of Papillary Thyroid Cancer

**DOI:** 10.3390/ijms26104646

**Published:** 2025-05-13

**Authors:** Michelle Carnazza, Danielle Quaranto, Nicole DeSouza, Augustine L. Moscatello, David Garber, Steven Hemmerdinger, Humayun K. Islam, Raj K. Tiwari, Xiu-Min Li, Jan Geliebter

**Affiliations:** 1 Division of R&D, General Nutraceutical Technology, LLC, Elmsford, NY 10523, USA; michelle.carnazza@gnt-us.com; 2 Department of Pathology, Microbiology & Immunology, New York Medical College, Valhalla, NY 10595, USA; dquarant@student.nymc.edu (D.Q.); ndesouza@student.nymc.edu (N.D.); humayun_islam@nymc.edu (H.K.I.); raj_tiwari@nymc.edu (R.K.T.); xiumin_li@nymc.edu (X.-M.L.); 3 Department of Otolaryngology, New York Medical College, Valhalla, NY 10595, USA; augustine.moscatello@wmchealth.org (A.L.M.); david.garber@wmchealth.org (D.G.); steven.hemmerdinger@wmchealth.org (S.H.); 4 Department of Dermatology, New York Medical College, Valhalla, NY 10595, USA

**Keywords:** thyroid, papillary thyroid cancer, recurrence, biomarkers

## Abstract

The thyroid is a vital endocrine organ that regulates metabolism, heart rate, respiration, digestion, body temperature, brain development, skin and bone maintenance, and reproduction and fertility. Thyroid cancer (TC) is the most common endocrine malignancy, with an estimate of 44,020 new cases in 2025. Incidence has been increasing, most notably at 4–5% per year in young adults. Papillary thyroid cancer (PTC), the most common TC subtype, accounts for approximately 80% of newly diagnosed TC cases. Furthermore, 2290 deaths are expected from the disease in 2025, with survival at over 98% with treatment. However, as PTC occurs most frequently in young women, recurrences are frequent and the 10-year disease-specific survival rate for advanced PTC is less than 50%. This narrative review aims to describe the current understanding of the thyroid gland, the incidence and subtypes of thyroid cancer, and specifically the diagnosis, prognosis, treatment, and recurrence of PTC. This is supplemented by the role of molecular pathways and biomarkers in PTC.

## 1. The Thyroid Gland: Anatomy and Function

The thyroid gland is a butterfly-like shaped vital endocrine gland at the front base of the neck. Two capsules cover the thyroid gland with loose connective tissue between them, allowing for thyroid movement and positioning when swallowing [1]. The thyroid contains two lobes spanning each side of the trachea connected by the isthmus, a small bridge of thyroid tissue. Thyroid tissue consists of follicular and parafollicular (“C”) cells. Parafollicular cells secrete calcitonin, which helps regulate the amount of calcium in the bloodstream [1]. Cuboidal follicular cells comprise the single thyroid epithelium layer and make up the spherical structural and functional units of the thyroid gland, the thyroid follicles. Follicular cells produce thyroid hormones thyroxine (tetraiodothyronine, T4) and triiodothyronine (T3) [2]. T4 is the main form found in blood circulation as T3 is produced in a much smaller quantity, at 80% and 20%, respectively; however, both travel to reach almost all nucleated cells in the body [3]. These fat-soluble, active thyroid hormones are carried by plasma proteins, with less than 1% circulating as unbound free hormone [4]. Most of T4 will be converted to the more potent T3 in the liver and other tissues where T3 acts, through deiodination, which is the removal of an iodine atom by deiodinases (DIO1 and DIO2) [3]. To make the thyroid hormones, the thyroid gland needs iodine, which is obtained through the diet. Iodine is transported into the follicular cell via the sodium–iodide symporter (NIS) [2]. Within these thyroid follicles are colloid, a reservoir rich in protein, especially thyroglobulin (Tg), a critical component of thyroid hormone synthesis. The iodination of Tg at tyrosine residues is catalyzed by thyroid peroxidase (TPO) in the presence of dual oxidase (DUOX)-generated hydrogen peroxide. The amount of thyroid hormone secreted is intricately regulated by the hypothalamus and pituitary gland, as part of the hypothalamic–pituitary–thyroid axis [3]. The hypothalamus senses the plasma concentration of thyroid hormones, and when low, releases thyroid-releasing hormone (TRH), which signals the pituitary to increase the thyroid-stimulating hormone (TSH) in circulation [2]. This increase in TSH binding to the TSH receptor (TSHR) on the thyroid follicular cells regulates and increases the conversion of thyroglobulin to T3 and T4 [5]. The thyroid hormones produced regulate metabolism, heart rate, respiration, digestion, body temperature, brain development, skin and bone maintenance, and reproduction and fertility [5].

An improperly functioning thyroid can impact the entire body. There are several types of thyroid diseases, and unfortunately, they are common—an estimated more than 5% of people in the United States have some type of thyroid disorder [6]. Thyroid diseases are classified as either primary or secondary, originating at the thyroid or pituitary gland, respectively [7]. To test thyroid function, serum TSH and serum-free T4 are often investigated [8,9]. The four main conditions that affect the thyroid include hypothyroidism, hyperthyroidism, goiter, and cancer. Endocrine disorders, namely hyperthyroidism and hypothyroidism, involve the over- and underproduction of thyroid hormones, respectively. Common causes are autoimmune disorders that target autoantigens including Tg, TSHR, and TPO, resulting in under- or overproduction of thyroid hormones [7]. The thyroid is the most vascularized tissue after the brain, making it highly susceptible to autoimmunity due to the extensive and constant exposure to the immune system. As with a vast majority of autoimmune disorders, prevalence is higher in women [8]. Grave’s disease is an autoimmune disorder whereby TSHR is bound and activated by agonistic autoantibodies circumventing the negative feedback regulation of thyroid hormones and results in hyperthyroidism [8]. Hyperthyroidism symptoms include anxiety, restlessness, tachycardia, heat intolerance, weight loss, and bulging eyes [7]. Available treatments include drugs decreasing iodine uptake, surgery, or radioactive iodine (RAI) ablation, followed by hormone replacement therapy (HRT) [7]. Hashimoto’s thyroiditis is an autoimmune disease whereby immune cells, including lymphocytes, infiltrate the thyroid, initiate robust CD4+ T cell-mediated responses, and eventually convert the thyroid into a lymph node [8]. This prevents thyroid cells’ ability to make thyroxine, and hence, the thyroid becomes underactive, evidenced by high TSH and low T3/T4 levels. Hypothyroidism symptoms are the exact opposite of hyperthyroidism, and these include depression, fatigue, bradycardia, weight gain, cold intolerance, and dry, itchy, and thinning skin [7]. For Hashimoto’s, the primary treatment is thyroid hormone replacement [7]. Goiter is a condition whereby the thyroid gland is enlarged, either entirely or through the development of thyroid nodules [10]. Goiter can present with hyper- or hypothyroidism, or euthyroid, with normal levels of thyroid hormones produced [7]. Treatment depends on the cause of goiter, but as with previously described disorders, treatments include surgery, RAI, and HRT [10]. It is important to distinguish this condition from thyroid cancer. It remains controversial, however, whether women with these thyroid disorders have a higher risk of thyroid cancer [11].

## 2. Thyroid Cancer: Incidence and Subtypes

As with all cells in the body, cells of the thyroid can undergo genetic mutations that, when accumulated, result in uncontrolled growth and the formation of a tumor. Thyroid cancer (TC) typically originates in follicular cells, except for medullary thyroid cancer, which derives from the parafollicular cells and only accounts for ~5% of TC cases [12]. TC is the most common endocrine malignancy and has been increasing in incidence in all age groups in the United States over the last few decades (~3% annually) [13]. This is especially evident in the 15–19-year-old age group over the last 25 years, with TC incidence surpassing central nervous system cancers and equaling leukemias, as TC rates have increased 4–5% per year in both young males and females [14,15]. It is estimated that 44,020 new cases of TC will be reported in 2025, with 2290 deaths [14]. Several types of thyroid cancer exist, with significant differences in growth rate and aggressiveness.

**Figure 1:**
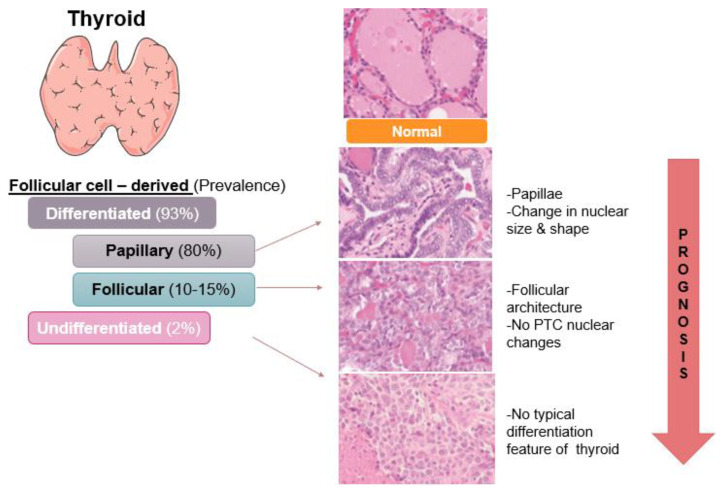
Follicular cell-derived thyroid cancers. Ranked by rate, PTC is the most prevalent follicular cell-derived thyroid cancer, followed by FTC and then undifferentiated (ATC). Prevalence also coincides with morphology changes and has an inverse correlation with prognosis. Histology images (magnification 200×) reprinted with permission from Ref: [18].

Cancer of the follicular cells is categorized into differentiated and undifferentiated. As implied, these are categorized based on the morphological characteristics that are retained from the normal tissue, and hence the functional characteristics (Figure 1). Papillary thyroid cancer (PTC) is a differentiated TC (DTC) and is the most prevalent (80%) [16]. Pathologically, PTC shows evidence of follicular cell differentiation with finger-like projections called papillae, which are central fibro-vascular stalks covered by a neoplastic epithelial lining. Additionally, PTC is characterized by the alterations of nuclear features, including enlargement, oval shape, elongation, inclusions, and grooves, and chromatin overlapping and clearing. Follicular thyroid cancer (FTC) is also differentiated but less prevalent, accounting for 10–15% of TC cases [16]. FTC is also characterized by the retained follicular architecture; however, it lacks the distinct PTC nuclear changes. Anaplastic thyroid cancer (ATC) exhibits no typical differentiation feature of the thyroid, and with decreasing differentiation comes a worsening prognosis [17]. Cytomorphological features include pleomorphism, including sarcomatoid, pleomorphic giant cell, and squamoid, and these can be alone or in combination. Histopathologically, ATC also presents with extensive necrosis and a high proliferation rate. These features are in part due to the structural and hence functional alterations that make the cancer cells resistant to current therapeutic modalities. This includes defects in iodine absorption, response to thyroid stimulating hormone, thyroglobulin expression, and thyroid hormone production. Therefore, ATC prognosis is extremely poor and aggressive, with high mortality and rapid disease progression resulting in an average survival time of 2–6 months after diagnosis.

**Figure 2:**
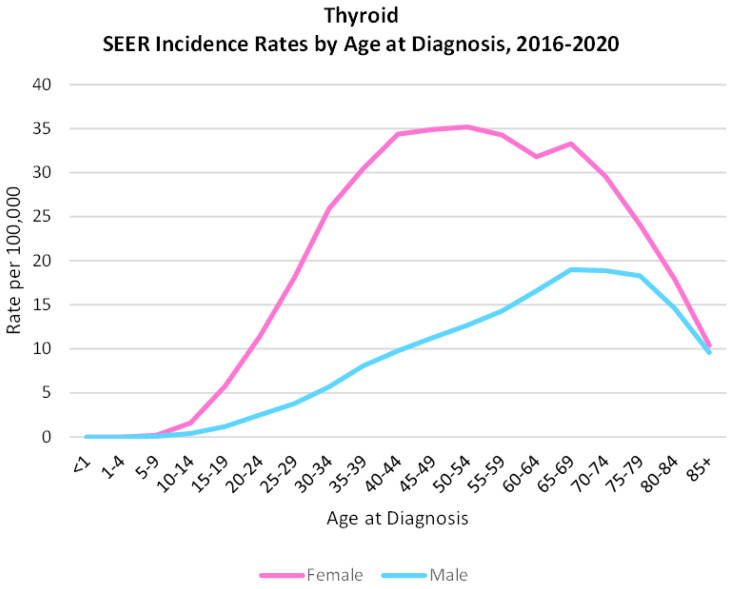
Thyroid cancer incidence rate by age at diagnosis among sexes, 2016–2020. Adapted from SEER (2024) [21].

Incidence and mortality rates are largely owed to PTC, as they constitute a significant fraction of TC cases. Risk factors for developing TC include radiation exposure, family history of thyroid or breast cancer, history of goiter, low iodine diet, and being a young female [19,20]. TC is the 7th most common cancer in females, accounting for 3% of all cancers, and the most common cancer in young adult females 20–29 years old [15,19]. It is evident that both sex and age are risk factors for development of TC, as at the onset of puberty, rates spike quickly in females and continue to rise until they peak and remain stable between the ages of 35 and 50 years old (Figure 2). This is in stark contrast to what is observed in males, as the female sex disparity peaks at almost 5:1 in younger adults 15–34 years old and then begins to normalize with age. On average, however, females are disproportionately affected at a rate of 3:1 over men. Additionally, incidence in males does increase with age; meanwhile, in females, it plateaus and begins to trend downward, equilibrating to an almost 1:1 ratio with males by advanced age. Age works against females in two stages, (1) hormones driving the cancer at a younger age then tapering down with age and subsequently and (2) the natural aging process whereby cancer risk increases in general. With that, people 55 years of age and older have an increased chance of metastatic spread and dedifferentiation into a more aggressive form of thyroid cancer.

The underlying cause for this sex disparity is poorly understood. Hypotheses proposed include body weight, body mass index, environmental and dietary factors, sex hormone receptor expression, and reproductive or menstrual status [22]. PTC incidence from 20–49 year olds coincides with the disparity in sex hormones between males and females, piquing interest in the role of sex hormones and their receptors. The increasing incidence in men with age corresponds with expected decreasing androgen levels. Therefore, a protective effect of circulating androgen and androgen receptors (ARs) could justify both the overall lower incidence in men and the increasing incidence with age. Additionally, the onset of menopause corresponds with a decrease in incidence in women, when sex disparity levels out to 1:1. It has been demonstrated that metastatic PTC is regulated by estrogen and the functional estrogen receptor (ER), enhancing the migratory, invasive, and proliferative phenotypes of cancer cells, as it does in other cancers such as breast cancer [23].

As indicated above, women present with a higher incidence of autoimmune diseases as well. It has been established that inflammation is a risk factor for the sex disparity seen in some cancers. With that, underlying inflammation may predispose women to thyroid cancer development. However, the underlying molecular mechanisms are poorly understood. Previous work suggested that AR activation attenuates Programmed Death Receptor Ligand 1 (PD-L1) expression in AR-responsive thyroid cancer cell lines, potentially though NF-κB (nuclear factor kappa-light-chain-enhancer of activated B cells) signaling, enhancing the susceptibility of cells to immune destruction [24]. Additionally, AR stimulation had an anti-proliferative effect in PTC through senescence induction [25,26]. Sex hormones have demonstrated tumor-modulating roles in other endocrine cancers including the breast and prostate, and there is emerging evidence of the role of sex hormones in non-endocrine-related cancers, including liver, lung, bladder, and colorectal cancers [27]. In prostate cancer cells, AR modulates proliferation and metastasis [28]. Additionally, there is evidence of androgens driving tumor progression in ovarian and breast cancer [29].

Also notable, women commonly present with less aggressive histological subtypes of PTC compared to men at diagnosis [30]. While expected incidence in 2025 for females is 3:1 that of males, the mortality rates are equal [14]. Therefore, it appears that the PTC that arises in men overrides the proposed protective effect of AR, yielding a more advanced cancer. Men have lower disease-free survival and higher mortality rates when they present with the disease [30]. Additionally, the rate of intermediate- and high-risk PTC is higher in males [30]. This coincides with other more adverse clinicopathological characteristics including angioinvasion, lymph node metastasis (LNM), and larger tumor size [30]. This suggests that PTC diagnosed in males is more aggressive which may worsen prognosis, and, therefore, treatment should be more aggressive. Further supporting the hormonal element of TC, ATC is more commonly seen in older populations (>65 years old) with no sex disparity [31]. Therefore, the sex disparity is largely due to the incidence of PTC.

## 3. Papillary Thyroid Cancer

PTC, the most common TC subtype, accounts for approximately 80% of newly diagnosed TC cases. PTC is one of the few cancers increasing in incidence in the US and globally. Most patients that are diagnosed with PTC present before 40 years of age. PTC is named for its papillae “finger-like” histology—a central fibrovascular stalk surrounded by a neoplastic epithelial lining of variable thickness and compositions [32]. Moreover, there are distinct nuclear features of PTC including elongation, enlargement, oval shape, inclusions, and grooves [33]. Grossly, PTC presents as an invasive neoplasm with poorly defined margins, a firm consistency, a granular white cut surface, and, possibly, calcifications [32,33]. There are histological variants beyond classical PTC that correlate with tumor aggressiveness and poor prognosis. Many variants have been characterized, including the more common follicular variant and tall cell variant [32,34]. Uncommon variants include the diffuse sclerosing variant, the solid variant, papillary thyroid microcarcinoma, the columnar cell variant, and the oncocytic variant [34,35]. Classically, PTC only affects one of two thyroid lobes, and ~60% of the time regional lymph node spread is seen at diagnosis; however, with appropriate treatment, it does not affect disease outcome [36]. While most cases are treatable, the early identification of patients requiring more aggressive treatment and follow-up compared to those having an indolent disease is an existing unmet need. Therefore, careful staging is critical for determining the extent of surgery, treatment options, and prognosis.

## 4. Diagnosis, Prognosis, Treatment, and Recurrence

In the early stages, most PTCs are asymptomatic. Eventually, as the tumor grows, one can notice a slow-growing lump in the neck, swelling of the neck, voice changes or hoarseness, trouble breathing or swallowing, a constant cough, or swollen neck lymph nodes. Unfortunately, routine blood tests do not help diagnose thyroid cancer as TSH, T3, T4, Tg, and calcitonin levels are commonly normal [37]. A routine wellness examination whereby a physician palpates the neck is commonly the initial identification of thyroid nodules, which is followed up with ultrasound imaging to determine number and size of nodules, if they are solid or fluid filled, and if there is spread to the cervical lymph nodes [36]. Additionally, non-palpable thyroid nodules can be identified incidentally through anatomic imaging studies. Thyroid nodules are discrete lesions radiologically different from the surrounding thyroid tissue and are very common, with some studies noting their prevalence up to 30%, but only a fraction are considered malignant [38]. Ultrasound guided fine needle aspiration biopsies (FNABs) are performed to determine if the nodule is malignant or benign; however, this is invasive and yields 40% indeterminate results, causing unnecessary surgery on benign nodules [39]. A shift in focus is necessary to address the overdiagnosis and subsequent unnecessary treatment and lifelong requirement for thyroid hormone replacement therapy and, hence, the needless burden on patients.

Histopathological features are the best prognostically, including capsular and vascular invasion, margin status, number and size of LNM, extranodal extension (ENE), and size of the metastasis [40,41,42] which directly correlate with prognosis. Prognostically, PTC is oftentimes curable, especially when diagnosed at an earlier disease stage. The current 5-year survival is over 98% with treatment, which is the highest of all cancers [14]. Importantly, cervical lymphatic spread does not worsen this good prognosis, still presenting an over 98% survival with treatment [36,43]. High survival is attributable to most subtypes being localized, slow-growing, and amenable to current therapeutic modalities [13,44]. Lymph node metastases are often present at diagnosis (>50%), in part due to the location, and the structure and function of lymphatics, as this system is essential for the transport of fluid from tissues. Taking advantage of this single layer of endothelial cells without a basement membrane, PTC cells do not need to be proteolytic to escape the thyroid, and the typical valves and junctions enable migration through them, much like leukocyte transmigration via chemoattraction. While LNM does not worsen the good prognosis of PTC, extrathyroidal extension (ETE) outside the fibrous thyroid capsule into surrounding tissue requires proteolytic degradation and penetration of the thyroid capsule.

**Figure 3:**
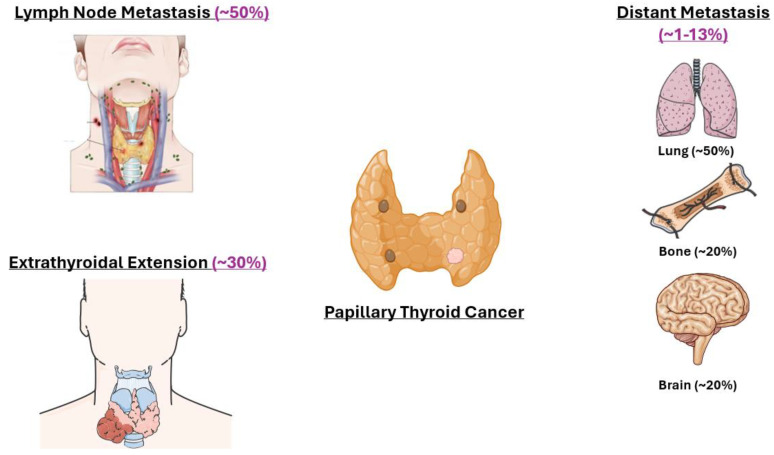
Clinical presentation of PTC. PTC is characterized by a high rate of lymph node metastasis (50%). Less frequently, extrathyroidal extension may occur (ETE; 30%). Distant metastases are rare (1–13%) but when present will largely spread to the lung (50%), bone (20%), or brain (20%).

As with all cancers, metastatic spread is a major concern, and ~10% of PTC patients may present with metastatic disease at initial presentation, decreasing survival to ~50% [45,46]. While distant metastases in PTC are uncommon, when metastasis occurs, common sites include the lungs (50%) and bone (20%) [47] (Figure 3). Furthermore, there are those more aggressive subtypes that dedifferentiate into more lethal TCs not adequately treated by current therapies.

Current therapeutic modalities for PTC include surgery, TSH suppression, and RAI ablation [48,49]. Use depends on the initial and ongoing stratification of risk and should be personalized to the needs and preferences of the patient. Fortunately, external beam radiation therapy (ERBT) and chemotherapy are not typically needed, as EBRT and chemotherapy are associated with substantial toxicity [50]. Additionally, radioiodine-refractory papillary thyroid cancer (RR-PTC) can be treated with tyrosine kinase inhibitors (TKIs); however, significant side effects and acquired resistance can occur [50,51]. Lenvatinib and sorafenib are currently approved for progressive RR-PTC [52]. However, the best treatment for PTC is total thyroidectomy, removing the entire thyroid. Specific instances will warrant removal of only the thyroid half with cancer, termed a hemithyroidectomy, with no requirement for lifelong thyroid hormone replacement. Hemithyroidectomy is considered when the tumor has not spread and the size is less than 4 cm. However, total thyroidectomy is recommended most because (1) most PTCs are multifocal, in more than one part of the thyroid, (2) post-operative RAI ablation is more effective, (3) follow-up for recurrence Tg blood test is more accurate, and (4) recurrence rate decreases, as this prevents cancer returning or spreading to the other lobe [53]. Unfortunately, removal of the entire thyroid requires taking HRT indefinitely. This signals to the brain that thyroid hormones are being produced and therefore suppresses TSH. HRT not only allows the thyroid to function normally, allowing for normal metabolism, but also prevents the high levels of TSH stimulating thyroid cancer and metastases to grow. Additionally, as LNM are commonly present at diagnosis, lymph node mapping before or during the surgery is conducted to assess the extent of involvement. Therefore, central cervical lymph node dissection is also common and can be conducted prophylactically in the absence of suspicious appearance [43,54]. However, there are higher risks of complications with prophylactic dissection including temporary or permanent hypoparathyroidism (low levels of parathyroid hormone) causing hypocalcemia (low levels of calcium in the blood) and temporary recurrent nerve injury; meanwhile, the recurrence rate remains the same [43,54]. In the event that lateral lymph nodes on the side of the neck are involved, a radial neck dissection could be performed at the time of thyroidectomy or afterwards.

RAI ablation is a daily pill with few side effects that takes advantage of the iodine uptake function of normal thyroid cells. RAI, therefore, ablates any remaining thyroid cells, benign and malignant, after initial surgery. Radioactive iodine uptake tests can quantify the predicted RAI accumulation during therapy through oral ingestion of low-dose RAI [55]. However, there is speculation of a stunning phenomenon that occurs with RAI therapy whereby the efficacy of the subsequent high-dose RAI is reduced, resulting in incomplete ablation [56]. Alternative isotopes are available that do not cause this phenomenon; however, they are limited and more expensive. Avidity to RAI may also be elucidated through serum Tg levels; however, autoimmune status, as described above, can confound the assessment [57]. Moreover, while RAI is a conventional and effective treatment for unresectable disease by exploiting thyroid follicular cells’ avidity for iodine, nearly ~50% of persistent, recurrent, or metastatic lesions lose the ability to take up radioiodine, having gone through a dedifferentiation process and losing *NIS* or *TPO* expression, resulting in RR-PTC [58,59]. This emphasizes the need for molecular markers of this more aggressive subtype of PTC.

Periodic follow-up is routine for thyroid cancer patients as thyroid cancer can return years after initial successful treatment—even if the thyroid was removed. Generally, blood tests every 6 months are conducted to assess Tg, calcitonin, and TSH secreted by the thyroid cells, as well as ultrasound of the neck to screen for nodules [40]. These circulating biomarkers, however, demonstrate inconsistency in their usefulness and do not distinguish between benign and malignant neoplasms or high- or low-risk tumors postoperatively. Recurrence can occur if microscopic cancer cells spread beyond the thyroid before thyroidectomy or small pieces of thyroid tissue were left behind during surgery [40]. Although prognosis of most cases is excellent, nearly 33% will develop recurrent disease and TC-related deaths [60].

**Figure 4:**
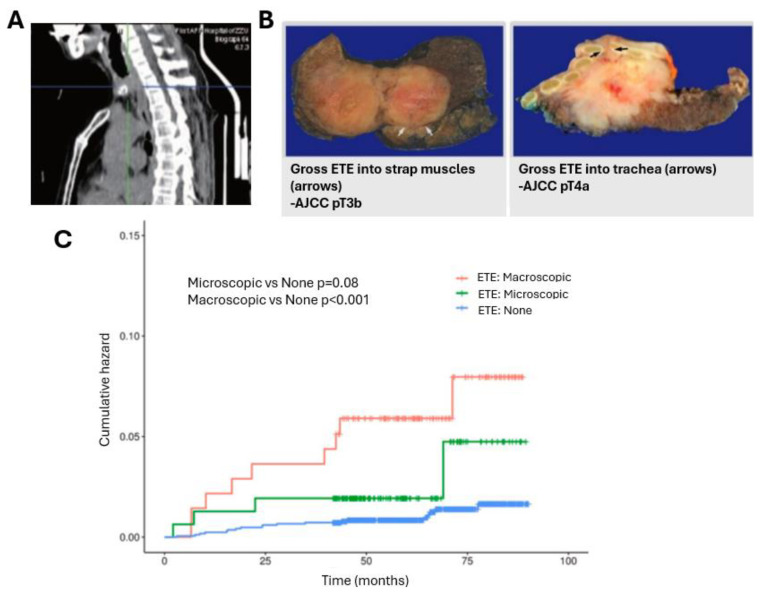
Extrathyroidal extension (ETE) in PTC. (**A**) Sagittal thyroid computed tomography scan shows that mass blocking most of the upper endoluminal trachea. Ref: [61] (**B**) Gross ETE into strap muscle (left) or adjacent organs (right) is considered AJCC pT3b and pT4a, respectively. Magnification: 40×. Reprinted with permission from Ref: [42] (**C**) Comparison of recurrence-free survival in PTC patients with different ETE classifications before propensity score matching. ETE, extrathyroidal extension. Reprinted with permission from Ref: [62].

Staging is pertinent to successful treatment strategy, follow-up, and quality of life for the patient. This is conducted radiologically and pathologically. The staging criteria most often used is the American Joint Committee on Cancer (AJCC) Tumor-Node-Metastasis (TNM) staging to estimate mortality. This system is classified by tumor size or extent, lymph node spread, and distant metastasis [40,47]. As such, this distinction beyond a stage T3a tumor comes from the presence of ETE [61], which only recently has gained significance in staging advanced stages (III vs. IV) [42]. Microscopic ETE, however, does not advance the stage to stage T3 or T4 [62] (Figure 4). This is regardless of lymph node involvement. Nearby lymph node involvement alone does not advance staging beyond Stage II as prognosis does not change in PTC but does determine if patients over 55 years are Stage I or Stage II. Age plays a critical role in prognosis, therefore, unlike other cancers, TNM staging of PTC takes age into account, adding a fourth staging criteria “A”, whereby Stage A1 is for patients under 55 years old and A2 is patients over 55 years old (Table 1).

### 4.1. Tumor

T1: less than 2 cm across and confined in the thyroid;

T2: between 2 and 4 cm and confined to the thyroid;

T3a: larger than 4 cm but confined in the thyroid;

T3b: larger than 4 cm but spread to the strap muscles around the thyroid;

T4a: tumor of any size that has grown extensively beyond the thyroid gland to nearby tissues in the neck, including the subcutaneous tissue, larynx, trachea, esophagus, or recurrent laryngeal nerve;

T4b: tumors of any size that have grown extensively beyond the thyroid gland toward the spine, into the posterior cervical fascia, or into major blood vessels nearby.

### 4.2. Node

N0: has not spread to any nearby lymph nodes;

N1: spread to any nearby lymph nodes.

### 4.3. Metastasis

M0: has not spread to distant sites;

M1: has spread to distant sites.

For PTC, M1 regardless of T or N stage warrants total thyroidectomy with or without RAI and local and systemic treatment [40]. In the event of M0 with a T3 or T4 stage and any N stage, total thyroidectomy, with or without lymph node dissection, and RAI are the primary treatment [40]. A PTC stage T2 with no lymph node involvement warrants total thyroidectomy with or without RAI or a lobectomy [40]. Meanwhile, stage T1 with N0 can warrant lobectomy or active surveillance of the tumor [40]. After 6 months to 1 year, response to treatment is evaluated, and when there is an excellent response, continued follow-up is recommended. With an indeterminate response, biochemical incomplete response, or structural incomplete response, the follow-up warrants additional treatment.

While TNM staging guides prognosis and treatment, these numbers do not take everything into account. The American Thyroid Association (ATA) highlights other important histopathological features to report including multifocality, surgical margins, number of lymph nodes examined and number with tumors with the size of the largest tumor-involved lymph node, extranodal invasion, and vascular invasion with the number of invaded vessels [36]. Furthermore, identifying the aforementioned variants by a pathologist to assess outcomes is recommended [36]. This can not only assist in guiding staging but also risk of recurrent or persistent disease and response to therapy [63]. Recurrence, years to decades after initial surgery, occurs in 25–35% of patients, the majority of which are now only in their 40/50 s. Recurrent PTC has an increased mortality rate of 60% [12,64,65,66,67,68].

The ATA classified recurrence risk as low, intermediate, and high based on metastasis-age-completeness of resection-invasion (ETE)-size (MACIS) Score [69,70] (Table 2). ETE occurs in ~30% of PTC patients and alone significantly decreases the favorable prognosis, as it strongly correlates to the local recurrence of PTC in the neck and the appearance of distant metastasis, which significantly decreases survival in PTC [41,47]. Therefore, it is critical to identify the extent of ETE as this will greatly influence diagnosis and therapeutic modality.

Surgically, removal of ETE that infiltrated into the strap muscles is warranted. However, infiltration into the esophagus, trachea, and inferior laryngeal nerve is debated. In an effort to mitigate overtreatment and affect patient quality of life, classification of recurrence risk has supplemented the decision to eliminate post-surgical RAI treatment in low-risk patients, as emergency evidence has contradicted its benefit [59]. Incomplete resection, especially in the instance of ETE, is a large issue. To allow the best outcome on survival, complete removal of cancer is vital. The goal of just eliminating cancer and preserving the function of the involved structure is rarely effective in macroscopic disease, resulting in persistence and a worsening prognosis, hence attributing to risk status. Of the intermediate- and high-risk patients that do develop recurrent or metastatic disease, 40% on average become resistant to conventional treatment and significantly decreases survival by 50% and 90% over five and ten years, respectively [59]. If RR-PTC occurs and patients are not amenable to surgery, ERBT may be used. Further, it is emphasized that risk stratification should continue to be assessed on follow-ups. As mortality rates, not incidence or survival, are better indicators of progress against cancer, it can be argued that recurrence, not 5-year survival, should be the indicator of PTC outcomes. It would be of clinical benefit to identify biomarkers associated with increased recurrence risk or aggressive cancer progression that could highlight a Stage I/II tumor with the propensity to become Stage III/IV. In this era of targeted and personalized therapy, it is critical to determine the RNA (ribonucleic acid) transcriptional profiles of primary tumors and invasive and metastatic sites. Thus, the identification of diagnostic and prognostic biomarkers that can guide surgery extent, therapeutic resistance, and recurrence risk is a critically important, unmet need.

## 5. Molecular Pathways and Characteristics of Papillary Thyroid Cancer

**Figure 5:**
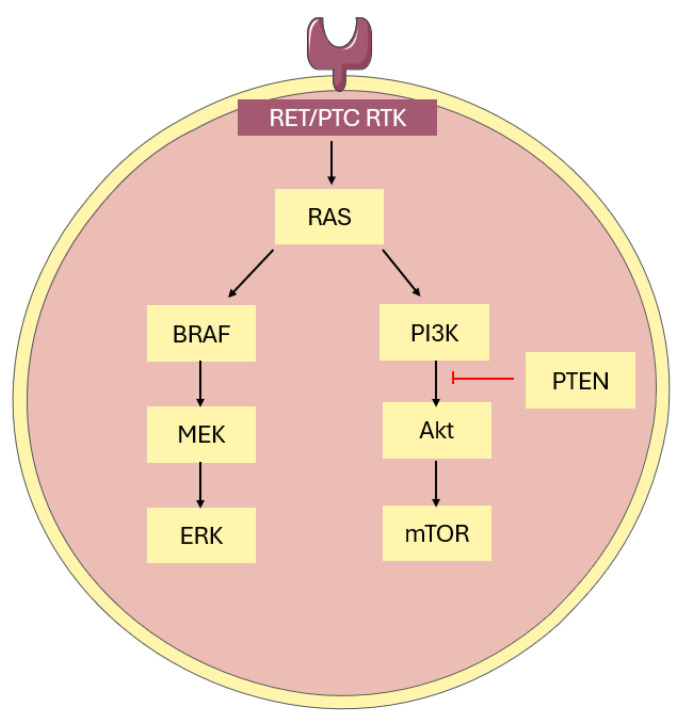
The molecular pathogenesis of papillary thyroid cancer. PTC driver mutations result in constitutive activation in the MAPK and PI3K/Akt/mTOR pathways, affecting cell proliferation and differentiation. Red lines represent inhibition.

It has been well established that PTC driver mutations result in constitutive activation in the MAPK (mitogen-activated protein kinase) and PI3K/Akt (phosphatidylinositol 3-kinase/protein kinase C) pathways, affecting cell proliferation and differentiation [12,71,72] (Figure 5). These mutations are termed “mutually exclusive” as it is rare to have multiple mutations, owing to the redundancy in their function [71]. Some of the highly relevant drivers include point mutations in *BRAF* (B- rapidly accelerated fibrosarcoma) and *N/K/H- RAS* (rat sarcoma virus) genes, *TERT* (telomerase reverse transcriptase), *TP53* (tumor protein 53), *EIF1AX* (eukaryotic translation initiation factor X-linked), *PIK3CA* (phosphatidylinositol-4,5-bisphosphate 3-kinase catalytic subunit alpha), as well as gene fusions of tyrosine kinases *RET* (Rearranged during Transfection), NTRK (neurotrophic tyrosine receptor kinase) 1, *NTRK3*, *ALK* (anaplastic lymphoma kinase), and *PAX8/PPARϒ* (paired box 8/peroxisome proliferator-activated receptor gamma-1) rearrangement and *MET* (mesenchymal epithelial transition factor) overexpression [12]. Epigenetic alterations including deoxyribonucleic acid (DNA) methylation and histone modification also appear to contribute to the clinical and behavioral factors of PTC. These genetic and epigenetic alterations have also been proposed for diagnostic/prognostic markers for indeterminate FNAB cytology, with the most popular being a gene panel comprised of *RAS*, *BRAF*, *RET/PTC*, and *PAX8/PPARϒ* [73,74].

BRAF is the most common oncogene reported to be involved in thyroid carcinogenesis, occurring in an average of 75% of patients [12,74]. Of these, about 67% harbor the specific point mutation T1799A whereby valine (V) is substituted for glutamic acid (E) at position 600 (V600E) [74]. Other BRAF point mutations, deletions, and gene fusions also exist. BRAFV600E results in constitutive activation of BRAF kinase activity, which activates the MAPK pathway and causes uncontrolled growth and spread of cancer cells by inhibiting differentiation and apoptosis. Therefore, BRAFV600E has been associated with more advanced clinical stage, higher prevalence of LNM, higher rates of disease recurrence, and shorter disease-free and overall survival [12,75]. Downstream, repression of thyroid-specific genes occurs which leads to de-differentiation, tumor progression, and more aggressive phenotypes. This suggests RR-PTC is due to the aberrant silencing of iodine-metabolizing genes due to this activation [76]. However, there are varied differentiation states and clinical behaviors of BRAF mutants, so it appears to clearly indicate a worse prognosis when in combination with other unfavorable mutations and clinicopathological characteristics of PTC and has therefore been suggested to not serve as standalone prognostic indicator. This point mutation is a target for specific BRAF inhibitors, including Vemurafenib and Dabrafenib, which show promise but as described above resistance can be developed.

RET is a receptor for glial-cell-line-derived neurotrophic factor. It is a thyroid-specific proto-oncogene encoding a transmembrane tyrosine kinase receptor that is rearranged in PTC, named RET/PTC. However, there are more than 19 different described rearrangements, resulting from the fusion of RET with different genes that allow for constitutive activation of the RET-tyrosine kinase domain, resulting in uncontrolled proliferation and survival through RAS-MAPK or PI3K/Akt cascades. Donor genes include *H4*, *CCD6*, *PRKAR1A*, *NCOA4*, *Golgas*, *TRIM24*, *TRIM33*, *KTN1*, *RFG9*, *ELK*, *PCM1*, *TRIM27*, and *HOOK3* [77]. However, *RET/PTC1* and *RET/PTC3* constitute up to 90% of all rearrangements, fusing with gene *H4* and *NCOA4*, respectively [12]. RET rearrangement prevalence is estimated to be 10–30% of PTCs; however, it is present in 45–60% of adolescents and children with PTC [78]. *RET* mutations can also cause multiple endocrine neoplasia, along with mutations in *MEN1* and *CDKN1B* [77].

RAS proteins are GTPases involved in MAPK signaling, regulating vital physiological processes. Activating mutations in H, K, and NRAS are found in many cancers, but thyroid cancer was actually among the first tumors identifying it [79,80]. Evidence suggests that NRAS is the most oncogenic to the thyroid of all three RAS isoforms [80]. RAS mutations are relatively rare in PTC with ranges in the literature from 0 to 10%, but they account for 30–45% of FTC, 20–40% of PDTC, and 10–20% of ATC [81]. RAS mutations also occur in benign follicular thyroid adenoma [79]. Thus, RAS mutations have minimal diagnostic specificity and sensitivity. RAS mutant tumors are typically not aggressive and yield good prognosis. Previous reporting of aggressive behavior was recently eradicated as this is through cooperative TERT mutations. Clinically, RAS mutations in conjunction with other characteristics are required to be relevant.

From the TCGA study, PTCs are classified by molecular subtypes BRAF-like (BL) and RAS-like (RL), whereby a tumor expresses transcription profiles resembling that of BRAF or RAS mutants [34]. These profiles differentially regulated MAPK and PI3K pathways producing large differences including in differentiation and histology. RL-PTCs are highly differentiated with follicular histology while BL-PTCs are less differentiated [34]. In addition, there are other well-defined molecular signatures elucidated by the TCGA datasets beyond the BRAF-RAS score (BRS), including the thyroid differentiation score (TDS) and proteomic ERK score [34,82]. The BRS is highly correlated to the TDS and ERK activation level. The BRS is calculated from 71 gene signatures, including RAS-like signatures ankyrin repeat domain 45 and malectin, to name a few [34]. BRAF-like genes include, among others, collagen 8 alpha 2 (*COL8A2*), fibronectin 1 (*FN1*), integrin alpha 3 (*ITGA3*), *MET*, and transforming growth factor beta receptor (*TGFBR*) 1 [34]. The ERK score is calculated from 52 MAPK-signaling pathway genes, including, amongst others, *MAP2K3* (MAPK kinase 2) and *MYC* (Myelocytomatosis Viral Oncogene) [34]. The TDS is calculated from 16 genes of thyroid function and metabolism. These genes are *DIO1*, *DIO2*, *DUOX1*, *DUOX2*, forkhead box E1 (*FOXE1*), GLI- similar family zinc finger 3 (*GLIS3*), NK2 homeobox 1 (*NKX2-1*), paired box 8 (*PAX8*), solute carrier family (SLC) 26A4, *SLC5A5*, *SLC5A8*, *TG*, thyroid hormone receptor (THR) A, *THRB*, *TPO*, and *TSHR* [34]. The TDS has previously been correlated with tumor grade, MACIS score, and risk of recurrence [34].

**Figure 6:**
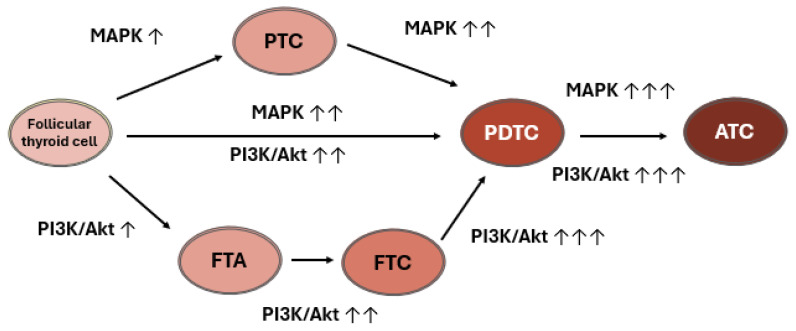
Thyroid cancer progression driven by MAPK and PI3k/Akt pathways. Genetic alterations leading to MAPK pathway activation primarily drives PTC development while PI3K-Akt primarily drives follicular thyroid adenoma and FTC. Accumulations of genetic alterations can strengthen both pathways’ signaling, with the potential to convert to the more aggressive poorly differentiated or undifferentiated TC. The increasing number of vertical arrows and color intensity of the ovals symbolize the increasing genetic alterations and signaling of the two pathways as thyroid tumorigenesis progresses.

It has been suggested that the relatively low overall density of somatic mutations could account for the indolent behavior of PTC. However, while its genomic landscape is relatively stable, aggressive PTC may occur, de-differentiating and becoming more lethal [75] (Figure 6). It is thought that with dedifferentiation, PTC gives rise to poorly differentiated thyroid cancer (PDTC) and ATC. As appropriate genetic alterations accrue in synergy with other molecular alterations, and MAPK and PI3K-Akt signaling intensifies, it is thought that the PDTC intermediate, and subsequently ATC, can develop [75]. MAPK and PI3K/Akt pathway activation results in downstream signaling of molecules including BRAF, MEK, AKT, and mTOR [83]. Together, these promote recurrence and mortality through the promotion of tumorigenesis and activating invasion and metastasis.

## 6. Biomarkers in Papillary Thyroid Cancer

The recurring contention above emphasizes the need for the identification of biomarkers or tumor markers that would guide and aid in cancer risk, screening, diagnosis, prognosis, treatment, and surveillance of various cancers, including PTC. Cancer is a diverse set of diseases, which highlights the need for precision medicine, based on not only the cancer type but the specific genes and proteins. This has been made apparent as not all patients with the same cancer are successfully treated with the same therapies. Hence, precision medicine utilizes anti-cancer drugs to target specific proteins that are involved in cancer growth and survival, in the hopes of preventing the proliferation of the cancer cells with minimal effect on normal cells [84].

Biomarker testing can be molecular, physiological, cellular, or image based. This can be conducted for patients with both blood cancer and solid tumors, looking at genes, proteins, and other biomolecules. Biomolecules, found in either the tissue or body fluid, can be present on or produced by cancer cells or normal cells in response to the cancer cells. Additionally, genetic testing can elucidate the risk of cancer susceptibility for those that are associated with germline genetic variations such as hereditary cancers [85]. The current workflow for novel biomarker identification, or the signature of several biomarkers, includes discovery, assay development and analytical validation, clinical validation, clinical utility, and then clinical implementation [85].

As genomic instability is a hallmark of cancer, most cancers result from the accumulating somatic mutations; therefore, common cancer biomarkers are single-nucleotide variants or variants of a few nucleotides and rearrangements [85]. Sequencing of the tumor upon biopsy can reveal the presence of the more prevalent and characterized variants or rearrangements in cancer, such as BRAF*V600E* or RET rearrangements, respectively [51,74]. Genes that are tumor-specific, mutated, or amplified or gene fusions can all serve as biomarkers. These alterations have effects at the DNA, RNA, and protein level. Additionally, epigenetic variants altering DNA, including methylation and histone modification, can also occur early on in tumorigenesis and can even be detected in plasma and serve as biomarkers [74]. Transcriptionally, messenger RNA (mRNA) and noncoding RNA (ncRNA) have demonstrated significant effects on carcinogenesis.

Tumor mRNA sequencing has identified differentially expressed genes in tumor vs. normal tissue and across other clinicopathological characteristics including stage and histological subtype. The detection of molecular cancer biomarkers is realized through polymerase chain reaction (PCR), next-generation sequencing (NGS), and microarrays [74,85]. While gene expression does not necessarily correlate with protein expression, proteins are more difficult to study. This is in part due to their complexity and sensitivity in addition to their post-translational regulation. Additionally, liquid biopsies contain normal proteins that are dominantly expressed, which can oftentimes mask protein modifications due to cancer [74]. Protein cancer biomarkers found in the tumor can be mutated, overexpressed, or harbor specific post-translational modifications. They also can be tissue-specific proteins that are found in body fluids at an increased concentration than normal. Commonly, these include cancer antigens, enzymes, hormones, and post-translational modifications including glycosylation [85]. Mainly, tumor proteins are assessed with the immunohistochemistry (IHC) of patient tissue biopsies and enzyme-linked immunosorbent assay (ELISA) of liquid biopsies [85]. Liquid biopsies are predominately non-invasive and include extra-tumoral samples including blood, urine and stool, and, less often, buccal swabs, cerebrospinal fluid, and sputum [85]. For blood samples, different parts can be used for biomarker testing including lymphocytes, circulating tumor cells, and nucleic acids, plasma, serum, or extracellular vesicles (EVs) [74]. However, liquid biopsy alone cannot replace tumor biopsy evaluation.

Tumor biopsies can also assess the presence of ncRNAs, which comprise both long ncRNAs (lncRNAs) and small ncRNAs, making up transcripts of greater than or less than 200 nucleotides in size, respectively. lncRNAs include long intergenic RNAs (lincRNAs), antisense RNAs, pseudogenes, and circular RNAs (circRNAs). lncRNAs regulate gene expression through alternative splicing, post-transcriptional modification of mRNA, and epigenomic alterations. lncRNAs can serve as scaffolds, bringing together multiple proteins, including the stabilization of nuclear structures or signaling complexes on chromatin. Additionally, lncRNAs can serve as guides for chromatin modifiers by binding target DNA or serve as molecular signals through the action of transcription factors and signaling pathways [86]. Furthermore, lncRNAs can act as decoys, titrating proteins, but also act to sponge miRNAs, pseudogenes, and splicing factors [86]. The tissue specificity of lncRNAs make them attractive targets, as does their demonstrated critical role in biological processes and regulation of various diseases. Furthermore, lncRNAs have been associated with tumor stage, metastasis, and survival, indicating their good potential in diagnosis and prognosis.

Small ncRNAs include small interfering RNAs (siRNAs), small nucleolar RNAs (snoRNAs), ribosomal RNAs (rRNAs), transfer RNAs (tRNAs), and miRNAs. miRNAs are approximately 20 nucleotides in length and function in post-transcriptional gene regulation through complementary binding with mRNA, resulting in mRNA slicing or translational inhibition and mRNA decay. miRNAs are transcribed from DNA into primary miRNAs (pri-miRNAs) and transported out of the nucleus to be processed to mature miRNAs by DROSHA and DICER in the cytoplasm. miRNAs can themselves be oncogenes and tumor suppressor genes (TSGs). Their small size and stability make them suitable biomarkers for samples of low RNA quality, with their expression being differentially expressed in both the tumor tissue and body fluids. In the blood, miRNAs are being studied as cancer biomarkers as they are the cargo of EVs and have been found to be more informative as a cancer biomarker [74,86]. The cross talk of lncRNAs with miRNAs highlights the regulatory role of the lncRNA-miRNA-mRNA axis. Here, lncRNAs act as competing endogenous RNAs (ceRNAs) whereby they sponge up miRNAs and prevent their binding to target mRNA. In cancer, the miRNA sponging function of lncRNAs has been implicated in regulating proliferation, apoptosis, growth, EMT, migration, and invasion [87]. Through downstream mRNA targets, suppression of miRNAs by lncRNAs altered pathways including Notch signaling, PI3K/Akt, and Wnt, to name a few [87]. This further highlights the value of the identification of clinically relevant coding and noncoding RNAs in cancer.

While modern diagnostic and therapeutic modalities yield a high rate of success, PTC management could see improvement through the identification of cancer biomarkers that are both sensitive and specific. For example, preventing over-aggressive surgical treatments for a relatively indolent PTC could prevent risks that outweigh rewards. Secondly, circulating Tg and TgAb levels are routine, but as indicated above, they do not provide ample preoperative value. And lastly, as described, FNAB has its limitations, with indeterminate results and the inability to adequately characterize follicular lesions [88]. This is important as thyroid neoplasms range from benign follicular adenoma to low-risk neoplasms to high-risk neoplasms to metastatic disease, and it would be beneficial to differentiate them as accurately, quickly, and non-invasively as possible. Ideally, biomarkers can supplement cytological examination preoperatively and/or serve in postoperative surveillance. NGS and publicly available datasets have enabled the identification of PTC biomarkers elucidating molecular mechanisms and aiding in diagnosis and prognosis. Some examples of potential ncRNA biomarkers of PTC include miR-222, miR-486, HOTAIR, and MALAT1, to name a few, which, through their interaction with their mRNA or miRNA target, demonstrated functions in proliferation, migration, invasion, and progression [89]. Work in our lab highlighted the potential role of MEG3 and FAM95C as negative and positive prognostic indicators in PTC, respectively [90]. Others have identified overexpression of protein-coding genes in thyroid tumors or patient serum, including but not limited to DICER1, EIF1AX, PDGFA, KLK7, KLK10, Galectin-3, and calprotectin, emphasizing their diagnostic value [91,92,93,94]. Circulating immunological markers have also been suggested to play a role in disease surveillance, including FAS and IFN alpha [88]. Increasing evidence also suggests the role of PD1/PD-L1 as a diagnostic, prognostic, and therapeutic marker of PTC, emphasizing a role in combination immunotherapy for RR-PTC and metastatic PTC [95,96,97,98]. Additionally, markers of the TME that have been proposed as TC biomarkers include platelet volume; Treg and TAM frequency in LNM; cancer-associated fibroblast marker expression including PDGFRB and vimentin in LNM; and a higher ratio of peripheral blood neutrophils to lymphocytes in larger tumors with higher recurrence risk [74,99]. However, it is important to note that most biomarkers are shared with other cancers, mitigating their specificity.

Molecular characterization of tumors is not required per clinical guidelines before starting TC treatment. Indeterminate nodules preoperatively warrants available molecular testing in the United States, including ThyroSeq, Afirma and Xpression Atlas, ThyGeNEXT, and ThyraMIR, and Rosetta GX [74]. While molecular testing is not currently a clinical guideline in RAI therapy determination, the *TERT* mutation co-occurring with *BRAF* or *RAS* mutations associates with RAI resistance [51]. Current methods to determine the RAI response have substantial weaknesses; therefore, biomarkers to predict responsiveness have been proposed. Furthermore, treatment with targeted drugs would benefit from screening for known molecular alterations, as PTC harbors more frequently targetable alterations; however, this is also not recommended in a clinical guideline [51]. This is important as some targeted therapies are only utilized when certain molecular characteristics are present; therefore, molecular testing would be required. With that, the identification of novel biomarkers that prevent unnecessary surgery and predict aggressiveness in PTC would provide immense value to the current marching orders of PTC diagnosis, prognosis, treatment, and surveillance.

## 7. Conclusions and Future Directions

Papillary thyroid cancer is often diagnosed as a small and localized disease, owing to this thyroid cancer subsets’ high survival and good prognosis. However, preoperatively it is difficult to reliably predict the risk for recurrence and mortality that may occur later in life, when patients are still only middle aged. BRAF mutations are common in PTC and have been identified as a key driver that also correlates with aggressive tumor behavior; however, they cannot predict outcomes alone. Therefore, advancement in the technology for molecular characterization of tumors is imperative for creating an individualized treatment plan that may guide prognosis and treatment strategies.

## Figures and Tables

**Table 1:** AJCC thyroid cancer staging.

AJCC Stage	Age	Description
Stage I	<55 (A1)	Any T, Any N, M0
	≥55 (A2)	T1, N0 or NX, M0
	≥55 (A2)	T2, N0 or NX, M0
Stage II	<55 (A1)	Any T, Any N, M1
	≥55 (A2)	T1, N1, M0
	≥55 (A2)	T2, N1, M0
	≥55 (A2)	T3a or T3b, Any N, M0
Stage III	≥55 (A2)	T4a, Any N, M0
Stage IVA	≥55 (A2)	T4b, Any N, M0
Stage IVB	≥55 (A2)	Any T, Any N, M1

A = Age; T = Tumor; N = Nodes; M = Metastasis.

**Table 2:** ATA disease persistence/recurrence risk stratification.

Recurrence Risk	Description
Low	No local or distant metastasis, the tumor is completely resected, no vascular invasion or invasion of locoregional tissues was observed, and the tumor does not have an aggressive histology
Medium	Microscopic invasion in the perithyroidal soft tissue, cervical lymph node metastasis is present, or the tumor has an aggressive histology or vascular invasion
High	Evidence of macroscopic tumor invasion, tumor resection was incomplete with gross residual disease, or distant metastases are present

## Data Availability

Not applicable.

## Abbreviations

The following abbreviations are used in this manuscript:

TC	Thyroid cancer
PTC	Papillary thyroid cancer
DIO	Deiodinase
NIS	Sodium–iodide symporter
Tg	Thyroglobulin
TPO	Thyroid peroxidase
DUOX	Dual oxidase
TRH	Thyroid-releasing hormone
TSH	Thyroid-stimulating hormone
TSHR	Thyroid-stimulating hormone receptor
RAI	Radioactive iodine ablation
HRT	Hormone replacement therapy
DTC	Differentiated thyroid cancer
FTC	Follicular thyroid cancer
ATC	Anaplastic thyroid cancer
AR	Androgen receptor
ER	Estrogen receptor
PD-L1	Programmed Death Receptor Ligand 1
LNM	Lymph node metastasis
FNAB	Fine needle aspiration biopsy
ENE	Extranodal extension
ETE	Extrathyroidal extension
ERBT	External beam radiation
RR-PTC	Radioiodine-refractory papillary thyroid cancer
TKI	Tyrosine kinase inhibitor
AJCC	American Joint Committee on Cancer
TNM	Tumor-Node-Metastasis
ATA	American Thyroid Association
MACIS	Metastasis-Age-Complete resection-Invasion-Size
MAPK	Mitogen-activated protein kinase
PI3K/Akt	Phosphatidylinositol 3-kinase/protein kinase C
BRAF	B- rapidly accelerated fibrosarcoma
DNA	Deoxyribonucleic acid
RL	Rat sarcoma virus-like
TDS	Thyroid differentiation score
